# Anti-Thymocyte Globulin (ATG)-Free Nonmyeloablative Haploidentical PBSCT Plus Post-Transplantation Cyclophosphamide Is a Safe and Efficient Treatment Approach for Pediatric Acquired Aplastic Anemia

**DOI:** 10.3390/ijms232315192

**Published:** 2022-12-02

**Authors:** Rong-Long Chen, Peng Peng Ip, Jy-juinn Shaw, Yun-Hsin Wang, Li-Hua Fan, Yi-Ling Shen, Nithila A. Joseph, Tsen-Erh Chen, Liuh-Yow Chen

**Affiliations:** 1 Department of Pediatric Hematology and Oncology, Koo Foundation Sun Yat-sen Cancer Center, Taipei 11259, Taiwan; 2 Institute of Molecular Biology, Academia Sinica, Taipei 115024, Taiwan; 3 School of Law, National Yang Ming Chiao Tung University, Hsinchu City 30093, Taiwan; 4 Department of Chemistry, Tamkang University, Tamsui, New Taipei City 251301, Taiwan; 5 Department of Pharmacy, Koo Foundation Sun Yat-sen Cancer Center, Taipei 11259, Taiwan

**Keywords:** anti-thymocyte globulin, nonmyeloablative haploidentical peripheral blood stem cell transplantation, post-transplantation cyclophosphamide, *SAMD9L* variant, severe aplastic anemia

## Abstract

Most cases of acquired aplastic anemia (AA) arise from autoimmune destruction of hematopoietic stem and progenitor cells. Human leukocyte antigen (HLA)-haploidentical nonmyeloablative hematopoietic stem cell transplantation (HSCT) plus post-transplantation cyclophosphamide (PTCy) is increasingly applied to salvage AA using bone marrow as graft and anti-thymocyte globulin (ATG) in conditioning. Herein, we characterize a cohort of twelve AA patients clinically and molecularly, six who possessed other immunological disorders (including two also carrying germline *SAMD9L* mutations). Each patient with *SAMD9L* mutation also carried an AA-related rare *BCORL1* variant or *CTLA4* p.T17A GG genotype, respectively, and both presented short telomere lengths. Six of the ten patients analyzed harbored AA-risky *HLA* polymorphisms. All patients recovered upon non-HSCT (n = 4) or HSCT (n = 8) treatments. Six of the eight HSCT-treated patients were subjected to a modified PTCy-based regimen involving freshly prepared peripheral blood stem cells (PBSC) as graft and exclusion of ATG. All patients were engrafted between post-transplantation days +13 and +18 and quickly reverted to normal life, displaying a sustained complete hematologic response and an absence of graft-versus-host disease. These outcomes indicate most AA cases, including of the *SAMD9L*-inherited subtype, are immune-mediated and the modified PTCy-based regimen we present is efficient and safe for salvage.

## 1. Introduction

Acquired aplastic anemia (AA), defined by persistent peripheral cytopenia accompanied by reduced bone marrow cellularity, can arise from diverse pathophysiological mechanisms, but it is mostly attributable to autoimmune-mediated destruction resulting in a profound deficit of hematopoietic stem and progenitor cells (HSPC) [1,2]. Most sporadic cases of acquired AA are labeled idiopathic, but subtypes attributed to inherited genetic variants or associated with specific immunological disorders, such as seronegative hepatitis, have been delineated [1,2,3,4]. In the era when the management of AA was limited to supportive care, patients with severe AA who received intensive transfusions and anti-infective treatments were still continuously threatened by fatal complications, such as bleedings and infections resulting in high mortality. For example, the reported chances of survival over two years were 36% and 25% in a domestic study and an earlier cohort study, respectively [5,6]. With the introduction of hematopoietic stem cell transplantation (HSCT) as well as non-HSCT measures which incorporate immunosuppression and stem cell stimulation approaches, the outcomes of current AA cohorts have improved substantially (reviewed in [2,7]). HSCT from human leukocyte antigen (HLA)-matched sibling donors has proven successful for pediatric treatment of acquired AA [8,9,10], indicating HSCT may correct the underlying immune dysregulation. HLA-matched unrelated HSCT as salvage of pediatric AA has also been associated with excellent survival but with a much higher frequency of serious graft-versus-host disease (GVHD) [11,12]. Since HLA-matched donors may not be readily available, the rapid and universal availability of haploidentical HSPC represents a viable graft source to salvage refractory AA. Given several potential treatment strategies, including granulocyte-colony stimulating factor-primed HSPC plus anti-thymocyte globulin (ATG), post-transplantation cyclophosphamide (PTCy), and ex vivo T cell depletion, haploidentical HSCT has been advocated as the salvage or even first-line treatment for pediatric AA [reviewed in [13,14]].

In this study, we adopt and modify the previously published protocol of haploidentical HSCT that deploys nonmyeloablative conditioning (containing rabbit ATG, cyclophosphamide, fludarabine, and low-dose total body irradiation), intensive GVHD prophylaxis (including PTCy, mycophenolate mofetil, and tacrolimus (a calcineurin inhibitor)), and that almost exclusively uses bone marrow as a graft source [15,16]. That original protocol elicited excellent GVHD-free survival without greater early toxicity for AA patients, but the substantial rate of graft failure resulting in up to 19% mortality remained worrisome [15,16]. Consequently, we modified the regimen by excluding rabbit ATG in conditioning and exclusively incorporating a high dose of freshly harvested peripheral blood stem cells (PBSCs) in an attempt to improve the salvage outcome of pediatric AA. We report the results from a cohort of 12 pediatric AA cases in our institute, with clinical and molecular characterizations emphasizing the efficiency and safety of the regimen in salvaging refractory pediatric immune-mediated AA, including in patients displaying hepatitis-associated and germline *SAMD9L* mutation-associated AA subtypes.

## 2. Results

### 2.1. Clinical Characteristics of the Patient Cohort at Transfer

Between 2013 and 2021, twelve pediatric patients (seven males and five females) with AA diagnosed at 2.5 to 19.5 years of age were transferred to our institute for salvage of various adverse events. Briefly, Cases 1–4 had minor symptoms but Cases 5–12 continuously experienced life-threatening bleedings and infections with or without iron-overload at transfer. Their clinical characteristics, treatments given before and after transfer, adverse events at transfer after treatments at previous hospitals, and AA outcomes after salvage are summarized in Table 1. Eleven patients were categorized as severe AA, whereas Case 1 was categorized as moderate AA. The severity and diagnostic criteria of AA according to peripheral hemogram and bone marrow cellularity were modified from the Camitta criteria [6,17] (Table 1). All cases had undergone bone marrow cytogenetic analysis at least once, which revealed normal karyotypes, apart from Case 11 whose bone marrow cytogenetics before HSCT revealed the 47, XY, +8 karyotype in 5 of 12 metaphases. AA treatments at previous hospitals included different combinations of immunosuppressive treatments, such as rabbit ATG, cyclosporine, and eltrombopag, as well as front-line PBSC transplantation (PBSCT) from an HLA-matched sibling for the severe AA in Case 6 (Table 1).

**Figure 1:**
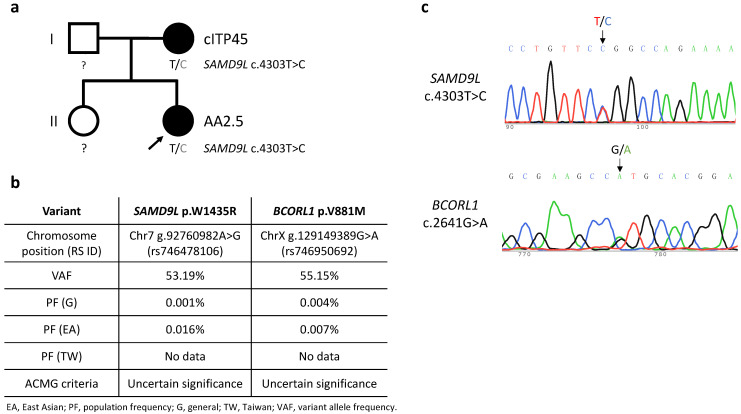
(**a**) Pedigree of the Case 2 family. Black shading represents carriers of the *SAMD9L* c.4303T>C mutation and presence of relevant disease phenotypes, i.e., cITP45 (chronic immune thrombocytopenia onset at 45 years of age) and AA2.5 (aplastic anemia onset at 2.5 years of age). Squares and circles represent males and females, respectively. (**b**) Characteristics of the detected germline *SAMD9L* and *BCORL1* variants. American College of Medical Genetics and Genomics (ACMG) criteria for variant interpretation encompass a five-tier classification: pathogenic, likely pathogenic, uncertain significance, likely benign, and benign. Data on population frequencies in Taiwan was sourced from Taiwan BioBank. (**c**) Sanger DNA sequencing of *SAMD9L* exon 5 on chromosome 7 and *BCORL1* exon 4 on chromosome X from peripheral blood cells taken from proband (Case 2) of the studied pedigree.

Among our patient cohort, six cases were assigned as idiopathic (Cases 4, 5, 7, 8, 10, 12), and they were transferred for salvage of relapsed/refractory AA at three months to five years after diagnosing AA. Cases 1, 2, 3, 6, 9, 11 were found in association with other immune disorders, including preceding inflammatory bowel disease (IBD), preceding hemophagocytic lymphohistiocytosis (HLH), post-immunosuppressive treatment alopecia areata, post-transplantation Evans syndrome, post-transplantation Graves’ disease, and preceding idiopathic hepatitis, respectively. Cases 2 and 9 were also identified as carrying a germline *SAMD9L* mutation [[18] and see Figure 1]. Detailed descriptions of the patients having other immune disorders are as follows.

Case 1 suffered severe and recurrent gastrointestinal disorders from 10 years of age and was diagnosed with IBD at 19 years of age when he began receiving intermittent anti-inflammatory treatments (mainly sulfasalazine and steroids). He was diagnosed with AA at 19.5 years of age and fulfilled the criteria for diagnosis of moderate AA at 20.5 years of age at transfer.

Case 2 suffered severe febrile illness and was diagnosed with HLH, which was treated by means of intravenous immunoglobulins, steroids, cyclosporine, and etoposide four months before being diagnosed with severe AA at 2.5 years of age. The patient’s severe AA completely responded to a rabbit ATG/cyclosporine regimen, but she developed thrombocytopenia (platelet count 48 × 10^9^/L) with menorrhagia after a documented parainfluenza B infection at 10.5 years of age, leading to a diagnosis of relapsed AA (Figure 1).

Case 3, though having responded to a rabbit ATG/cyclosporine regimen and being subjected to a tapering-down CSA dosage, developed alopecia areata at transfer.

Case 6, at transfer, was diagnosed with post-transplantation Evans syndrome two years after successfully undergoing PBSCT from her HLA-matched brother.

Case 11, at four years of age, was diagnosed with severe AA five months after an episode of severe and acute hepatitis (peak total and direct bilirubin of 5.43 and 4.24 mg/dL, respectively; aspartate and alanine transferase 2380 and 1386 U/L, respectively), but with negative etiologic surveys for hepatitis A/B/C and thus was assigned as a case of hepatitis-associated AA.

### 2.2. Analyses of Genetic Variants and Telomere Length

**Figure 2:**
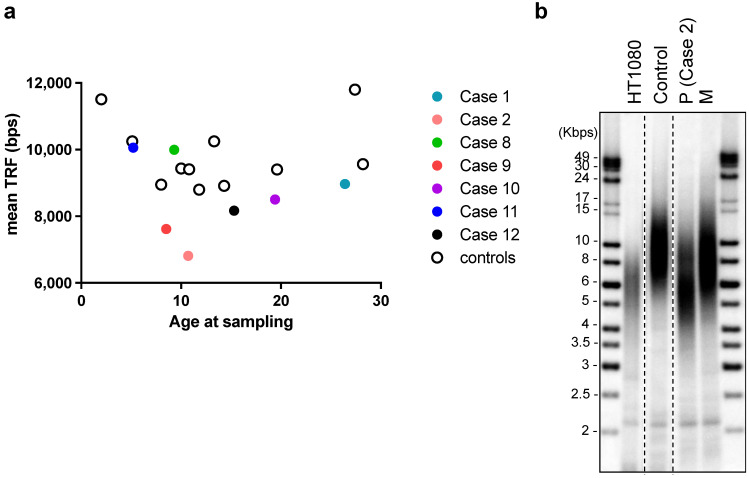
Telomere length analysis using terminal restriction fragment assay of DNA from leukocytes of: (**a**) Cases 1, 2, 8–12 in this cohort and age-matched controls (no diagnosis of AA); and (**b**) Case 2 patient (P) at diagnosis of aplastic anemia (age: 10.7 years old), Case 2′s mother (M, age: 48 years old), a healthy control of similar age to the patient (10.8 years old), and from HT1080 fibrosarcoma cancer cells. DNA samples were run in the same gel, which has been cropped for illustrative purposes.

Six patients (Cases 1, 2, 4, 8, 9, 12) were subjected to whole-exome sequencing. Two patients (Cases 2 and 9) were identified as carrying missense *SAMD9L* variants among a panel of genes associated with inherited bone marrow failure at an allelic balance consistent with a germline origin and with a variant allele frequency (VAF) of 53.19% (SAMD9L p.W1435R) and 54.04% (SAMD9L p.C1267F), respectively, and confirmed by Sanger’s sequencing (Case 2: Figure 1c, Case9: [18]). No other significant germline variants related to inherited bone marrow failure syndromes or telomeric disorders were identified. Mutation of *BCORL1* (BCORL1 p.V881M) was also detected in Case 2 (Figure 1c). We did not identify any other variants from the gene panel of AA-associated clonal hematopoiesis with indeterminate potential [19,20,21]. Leukocyte telomere lengths of seven of the AA patients, as well as the mothers of Case 2 and Case 9, respectively (both carriers of germline *SAMD9L* mutations), and age-matched controls were determined (Figure 2a). The two *SAMD9L*-mutation-associated AA patients, i.e., Case 2 (Figure 2b) and Case 9 [18], displayed shorter telomere lengths compared to age-matched controls.

### 2.3. Analysis of AA-Susceptible HLA and Other Single Nucleotide Polymorphisms (SNP)

We also checked for the presence of reported AA-susceptible *HLA* (n = 10, encompassing Cases 1–2, 4, 6–12) (Table 2) or non-*HLA* (n = 6) variants. Notably, AA-risky alleles of HLA-A*02, *HLA-DRB1* including *15 and *15:01, and HLA-DQB1*06:02 were present in six of the ten patients we analyzed [22,23] (Table 2). An AA-susceptible SNP (rs1042151 A > G, encoding *HLA-DPB1* Val76) reportedly associated with an increased risk of AA solely among Europeans [23,24] was not identified among our patient cohort. However, we did identify HLA-DRB1*03:01/*13:01 (Case 11) and *14 (Case 4), which are reportedly protective against AA [22]. Case 4 carried both AA-risky and -protective *HLA* alleles, whereas Case 11 (i.e., the patient suffering a hepatitis-associated AA subtype) only carried AA-protective alleles. For the six patients that underwent whole-exome sequencing of whole blood, we also assessed for the presence of other AA-risky non-*HLA* SNP, including telomeric repeat binding factor 1 (*TERF1*) (rs3863242) [25], *CTLA4* (rs231775) [26], *PDCD1* (rs36084323) [27], *IFNG* (rs2430561) [28], *FAS* (rs1800682) [29], and *FOXP3* (rs3761548) [30]. The only one that was detected was the *CTLA4* p.T17A (rs231775) SNP, with five of the six cases carrying the AG genotype and Case 9 carrying the GG genotype that has been reported as AA-risky in Chinese Han [26] but not in Caucasians [31].

### 2.4. Response to Salvage Treatments

Salvage responses to AA treatment were assigned as either complete response (hemoglobin ≥ 10 g/dL, absolute neutrophil count ≥ 1 × 10^9^/L, and platelets ≥ 100 × 10^9^/L) or very good partial response (hemoglobin ≥ 8 g/dL, absolute neutrophil count ≥ 0.5 × 10^9^/L, and platelets ≥ 50 × 10^9^/L) according to the North American Pediatric Aplastic Anemia Consortium definition [17,32]. All patients had attained sustained complete response at the latest follow-up, except for Case 2 who, because of her germline *SAMD9L* mutation and recurrent thrombocytopenia, was assigned to a watch-and-wait strategy and became asymptomatic (with platelets fluctuating between 64 and 98 × 10^9^/L, as well as displaying normal cytogenetic karyotypes upon repeated bone marrow analyses), compatible with a very good partial response for over three years since recurrence (Table 1). Case 1, suffering IBD-associated AA, responded to cyclosporine treatments for 22 months and retained a status of complete response for more than four years after cyclosporine had been discontinued. The alopecia areata presented by Case 3 at transfer improved upon increasing the cyclosporine dosage plus topical application of pregaine^®^ clear gel shampoo, and her AA status remained as complete response over eight years after gradually tapering off cyclosporine for an additional eight months after transfer. As a case of relapsed AA six months after discontinuing cyclosporine, Case 4 completely responded to re-institution of cyclosporine and was gradually tapered off it for a further 30 months, remaining in complete response status for over four years after cyclosporine was ultimately discontinued. Cases 5 and 6 were both in complete response status for their AA for over eight years after HLA-matched unrelated bone marrow transplantation and sibling PBSCT, respectively. The post-transplantation Evans syndrome of Case 6 required intermittent immunomodulatory treatments—including intravenous immunoglobulins, prednisolone, cyclosporine, and mycophenolate mofetil—spanning seven years, and the patient has now been disease-free for over two years after drug discontinuation.

**Figure 3:**
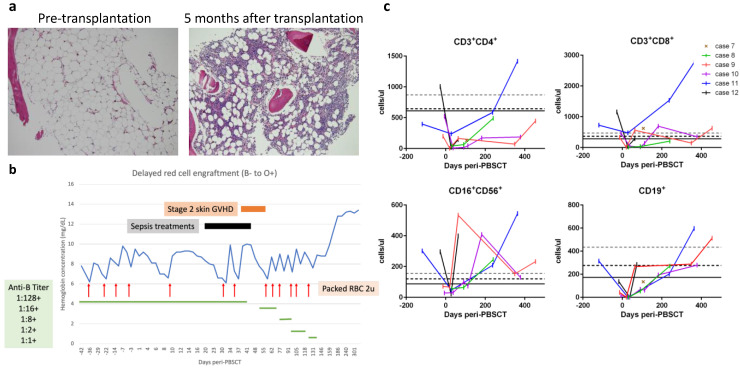
(**a**) Representative bone marrow biopsy specimens from Case 7 (10× objective, ×100 magnification; hematoxylin and eosin stain) illustrating pre-transplantation hypocellularity (left image) compared to recovered cellularity five months after transplantation (right image). (**b**) The time-course of delayed red cell engraftment of Case 8. (**c**) Reconstitution of lymphocyte subsets. Grid lines at Y-axis represent the 10th percentiles of lymphocyte subsets in healthy 2–6-year-old (grey dashed line), 6–12-year-old (black dashed line), and 12–18-year-old (black solid line) controls.

The remaining six cases (Cases 7–12) were transferred with refractory severe AA but lacked available HLA-matched donors; yet, they were eligible for haploidentical PBSCT. All these patients signed an institutional review board-approved informed consent form before undergoing PBSCT. The peri-transplantation characteristics of these six patients are summarized in Table 3. The time intervals from AA diagnosis to haploidentical PBSCT were three months to five years. The haploidentical donors (HLA-mismatched at 4–5 of 10 loci between the patient and the donor) included a patient’s father (n = 4), mother (n = 1), or brother (n = 1). The ABO/Rh blood types for the donors and patients are shown in Table 3, with the most notable case being Case 8 who possessed both major ABO and minor Rh incompatibilities. All these patients received a modified reduced-intensity conditioning protocol that included pharmacokinetic-guided fludarabine, cyclophosphamide, and low-dose total body irradiation but without rabbit ATG ([15,16] and described in Materials and Methods). On day 0, the patients received freshly prepared PBSCs containing higher doses of total nucleated cells (9.8 to 19.8 × 10^8^ cells) and CD34^+^ cells (8.8 to 16.6 × 10^6^ cells), as well as higher numbers of CD3^+^ cells (0.67 to 5.64 × 10^8^ cells) per kg body weight (Table 3). For GVHD prophylaxis, patients received PTCy followed by cyclosporine and mycophenolate mofetil, as described in the Materials and Methods. Other supportive measures for organ protection and prevention of infection are also described in the Materials and Methods. All six patients developed fever between post-transplantation days +1 and +3 that lasted for 1–5 days but were resolved soon after PTCy administration and without hypotension, oxygen requirement, or significant organ toxicities compatible with Grade 1 cytokine release syndrome [33]. Neutrophils were engrafted between days +13 and +18 with 100% donor chimerism achieved thereafter in all six patients (Table 3). Improved bone marrow cellularity was documented in Case 7 (Figure 3a). No transfusions were required after day +25, except for Case 8 displaying ABO/Rh incompatibility, who exhibited delayed red cell engraftment and required red cell transfusions until day +131 (Figure 3b). Lymphocyte subsets were reconstituted in a timely fashion in all six patients (Figure 3c). Moreover, almost all reached the milestone of early CD4^+^ immuno-reconstitution of 50 CD4^+^ T cells per μL by day +100, which is significantly associated with better survival and lower transplantation-related mortality [34]. The only exception was a slight delay for Case 10, who presented with 44 and 172 CD4^+^ T cells per μL on days +112 and +182, respectively. Case 10 also suffered peri-transplantation catheter wound-associated mixed infections, including methicillin-resistant *Staphylococcus epidermidis* and *Bacillus* species plus positive serum aspergillosis antigen tests, which were well controlled by treatments with multiple antimicrobial drugs. Otherwise, any instances of peri-transplantation febrile events for the six PBSCT patients were mild and easily controlled. Cases 6 and 7 had self-limited grade I/II skin GVHD, but none of the patients developed grade III/IV acute or chronic GVHD. Reactivation of cytomegalovirus without significant disease was documented in Cases 7 and 10 but was suppressed upon administering valganciclovir orally for four weeks. None of the patients developed Epstein-Barr virus-associated post-transplantation lymphoproliferative disorders. One notable complication was occurrence of Graves’ disease in Case 9, which has been reported separately [18]. All six patients reached sustained complete response between days +17 and +158: Cases 7, 9, and 11 reached complete response within a month; the delayed complete response of Cases 8, 10, and 12 could be attributable to ABO/Rh incompatibility, severe infections, and menorrhea rebound after estrogen withdrawal, respectively.

## 3. Discussion

Autoimmune-mediated recognition of specific epitopes presented on HSPCs by auto-reactive T cells to give rise to AA is a plausible explanation of its pathophysiology [35,36]. Evidence consistent with such an autoimmune-mediated mechanism is apparent among our cohort of AA patients. First, the patients displayed clinical associations with other autoimmune or immunoregulatory disorders, including IBD, HLH, alopecia areata, Evans syndrome, Graves’ disease, and immune-linked hepatitis (Cases 1, 2, 3, 6, 9, and 11, respectively). Second, markers of immune escape or advantage, such as the trisomy 8 karyotype and *BCORL1* missense variant, were identified in Case 11 and Case 2, respectively. Third, we detected previously characterized AA-risky *HLA* polymorphisms (in six of ten assessed patients), as well as the *CTLA4 p.T17A* GG genotype (in one of six patients). Among the six patients subjected to whole-exome sequencing, the *BCORL1* variant in Case 2 was the only molecular determinant identified by sequence analysis from a panel of genes linked to clonal hematopoiesis of indeterminate potential. Carriers of *BCORL1* mutation, such as *PIGA* and *BCOR*, exhibit a lower risk of progressing to secondary myeloid neoplasms, and thus, this variant could represent a marker of immune selection without being a significant driver of myeloid malignancy [37,38]. We also observed both *SAMD9L* mutation-associated AA patients (Cases 2 and 9) not only showed evidence of immune-mediated pathophysiology resulting in AA but also presented with significantly shorter telomeres upon presenting with cytopenia. As demonstrated for Case 9 [18], telomere length deficiency could be corrected upon successful haploidentical PBSCT.

HSCT has long been documented as effectively correcting the underlying immunodysregulation associated with AA, but graft failure and GVHD remain significant complications. Given technological advancements, as well as rapid and universal donor availability, haploidentical HSCT is being applied more frequently to treat AA [13,14]. Recent administration of a haploidentical bone marrow transplantation protocol with intensive GVHD prophylaxis comprising rabbit ATG, fludarabine, and PTCy markedly diminished the risk of GVHD, but the rate of graft failure remained unacceptably high [15,16]. Here, we report preliminary salvage results for a pediatric AA cohort, emphasizing our modification of the PTCy-based haploidentical HSCT protocol that encompassed removing rabbit ATG from conditioning, as well as the use of high-dose non-cryopreserved PBSCT, which resulted in efficient engraftment and safely corrected pediatric AA.

The introduction of PTCy on days +3 and +4 after HSCT, pioneered by the Johns Hopkins group, has greatly improved outcomes of haploidentical HSCT, resulting in low incidences of GVHD and non-relapse mortality [39] that may be attributable to PTCy-mediated protection through deletion of adoptively transferred alloreactive T cells and rapid preferential recovery of FoxP3^+^ regulatory T cells [40]. A report from the EBMT Severe Aplastic Anemia Working Party indicated unmanipulated haploidentical transplantation with PTCy to treat refractory AA patients elicited encouraging outcomes with satisfactory GVHD-free survival [41]. Given that graft failure still contributes most of patient mortality using the current PTCy-based protocol [15,16], further improvements are needed. A previous study deploying PTCy-based haploidentical HSCT reported patients who received augmented conditioning and those who received higher doses of bone marrow CD34^+^ cells exhibited greater event-free survival [42]. Since engraftment failure (~70%) following haploidentical HSCT for AA has long been a major impediment to successful treatment [43], we have been working to modify this protocol to further improve the outcomes for pediatric patients with refractory severe AA.

ATG has been established as a standard immunosuppressive agent for AA, including for pediatric patients, with ATG products derived from an equine source proving more efficacious than those from rabbit [44,45]. Nevertheless, supplies of equine ATG are limited and are not available in many countries. ATG, mainly from rabbit, is also extensively deployed in conditioning treatments for AA before HSCT [8,9,10,11,12]. However, exposure to rabbit ATG during pediatric HSCT pre-conditioning may result in delayed immune reconstitution post-transplantation due to the long half-life of ATG, which may also interact with PTCy, as determined by pharmacokinetic measurements [46]. In a report on haploidentical HSCT and PTCy to treat severe AA from the EBMT Severe Aplastic Anemia Working Party, no benefit of ATG in reducing GVHD or improving survival was determined. Indeed, diminished engraftment was observed among the patients treated with ATG [41]. Thus, it may be beneficial to remove ATG from haploidentical HSCT pre-conditioning with PTCy for AA. Similarly, we targeted a fludarabine cumulative concentration-time curve (AUC) of ~20 mg·h/L (range 17.5–22.5 mg·h/L) using a pharmacokinetics model given an association of fludarabine overexposure with impaired immune reconstitution or underexposure with increased graft failure and transplantation-related mortality has been reported [47].

PBSCs as graft source, compared to bone marrow, may provide a higher dose of CD34^+^ cells, which has been linked with greater event-free survival following PTCy-based haploidentical HSCT [42]. Outcome analyses by the European Society for Blood and Marrow Transplantation for both MSD and unrelated donor HSCT have indicated that bone marrow grafts are preferable for pediatric severe AA patients, resulting in lower rates of chronic GVHD and overall mortality, as well as better survival when compared to PBSCT [48,49]. However, when combined with PTCy, haploidentical PBSCT has proven promising in terms of low incidences of GVHD, approaching rates following haploidentical bone marrow transplantation [50]. Since PTCy-based haploidentical PBSCT for treating different hematologic diseases does not appear to be detrimental in terms of either GVHD or engraftment rates, this approach could represent a valid alternative to bone marrow transplantation in PTCy-appropriate contexts [51,52]. PTCy-based nonmyeloablative haploidentical PBSCT has been successful in rescuing refractory severe AA patients without increased rates of GVHD [53], and two patients who developed secondary graft failure after haploidentical bone marrow transplantation for AA were successfully salvaged by means of haploidentical PBSCT from different donors [54]. Here, we also used freshly prepared non-cryopreserved PBSCs for transplantation since a previous study reported higher rates of graft failure and mortality upon transplantation with cryopreserved rather than non-cryopreserved grafts [55].

Among our patient cohort, we applied haploidentical PBSCT to salvage all the pediatric patients displaying refractory severe AA but who lacked available HLA-matched donors. We found this strategy to be efficient for all those patients, including one each of germline *SAMD9L* mutation-associated and hepatitis-associated AA subtypes. Anticipated complications, such as graft failure, GVHD, cytokine release syndrome, organ toxicities, or chronic viral reactivation disorders, were absent or minor. Major complications in the cohort included delayed red cell engraftment for Case 8, post-transplantation Graves’ disease for Case 9 (discussed in [18]), and significant catheter-associated infections for Case 10. The prolonged persistence of anti-B isohemagglutinins in Case 8 was associated with the delayed red cell engraftment that may arise after major ABO-incompatible nonmyeloablative HSCT [56]. It has been suggested previously that ABO-compatible donors are a better option if several haploidentical donors are available because the associated requirement for prolonged transfusions substantially enhances various risks, including iron overload, alloimmunization, and transfusion reactions [57]. Severe infections are among the major causes of morbidities and mortalities arising from severe AA, but timely immune reconstitution after haploidentical PBSCT, as achieved here for our patient cohort, can greatly limit infection-linked complications.

The study is limited by the small case number enrollments as the single-institute pilot trial of haploidentical HSCT has been designed mainly for the salvage of transferred life-threatening refractory cases of pediatric aplastic anemia. Our clinical and molecular characterizations provide evidence of HSPC destruction through autoimmune-mediated mechanisms in our cohort of pediatric AA patients. We highlight our ATG-free, PTCy-based haploidentical PBSCT protocol exerts adequate immune ablation on recipients, provides sufficient donor HSPCs, and effects timely immune reconstitution post-transplantation, together representing an efficient and safe platform for salvaging refractory pediatric AA. Given the preliminary success, future studies may extend the applications to broader indications, for example, in frontline setting for pediatric aplastic anemia with unavailable HLA-matched sibling donors.

## 4. Materials and Methods

### 4.1. Identification of Variants by Whole-Exome Sequencing (WES)

Informed consent was explained and obtained from six patients and their parents prior to this study. Peripheral blood from the patients and their parents was collected before the patients underwent PBSCT. Genomic DNA from whole blood was isolated using a Wizard genomic DNA purification kit (Promega, Madison, WI, USA) for WES. A DNA library was prepared using Agilent SureSelect human all exon V8 (Santa Clara, CA, USA), and sequencing was performed using a NovaSeq 6000 system (Illumina, San Diego, CA, USA). The sequencing data were aligned to the GRCh37 (hg19) reference genome to identify genetic variants. High quality variants which passed the quality filters of average base calling quality (Phred score) >=30 and the total number of reads >=40 were selected for analysis. Rare variants with a population frequency <0.05% in the Genome Aggregation Database (GnomAD) and Taiwan BioBank database were further classified into pathogenic, likely pathogenic, uncertain significance, likely benign, and benign using ACMG guidelines [58]. Furthermore, we searched for low-frequency variants (variant allele frequency from 1%) in genes known to influence clonal hematopoiesis of indeterminate potential [19,20,21]. Mutations were verified by Sanger sequencing. A fragment of the *SAMD9L* gene containing the *SAMD9L* c.4303T>C mutation (Case 2) was amplified by PCR using 5′-GTCTAAAGCCCAACTCCAAG-3′ and 5′-GACCCT TCCTTTTGCCCAG-3′ primers. A fragment of the *BCORL1* gene containing the *BCORL1* c.2641G>A mutation (Case 2) was amplified by PCR using 5′-ATGCCCCTTGATCTGTCCTC-3′ and 5′-CGCCTTCCATCTTAGGAGTGCTG-3′ primers. The forward primer of each fragment was used for Sanger sequencing.

### 4.2. Determination of HLA Haplotypes and SNP

Next-generation sequencing-based *HLA* genotypes were extracted from donor and patient WES data using the HLAscan application [59]. In brief, WES sequence reads were aligned with human genome reference hg19 (UCSC browser). Each read in the aligned results was classified according to *HLA* class genes using the HLAscan application and exon sequences from the ImMunoGeneTics project (IMGT)/HLA database (https://www.ebi.ac.uk/ipd/imgt/hla/, accessed on 4 December 2019). Reads were aligned with exons 2, 3, 4, and 5 of *HLA* class I genes, and exons 2, 3, and 4 of *HLA* class II genes. Allele types were then determined based on the numbers and distribution patterns of the reads for each reference target.

### 4.3. Terminal Restriction Fragment (TRF) Assay

Leukocyte telomere length was analyzed by means of terminal restriction fragment assay, as described previously [60]. In brief, leukocyte genomic DNA was digested by *Rsa I* and *Hinf I* restriction enzymes and then resolved by pulsed-field gel electrophoresis. Telomeric DNA was detected by in-gel hybridization using a [^32^P]-labeled telomeric probe. Mean TRF values were determined using TeloTool software [61].

### 4.4. Conditioning Regimen and GVHD Prophylaxis for Haploidentical PBSCT

The conditioning treatments consisted of intravenous (i.v.) fludarabine (dose determination using a validated pharmacokinetic model [18,62] according to body weight and serum creatinine with an area under the curve (AUC) of between 17.5 and 22.5 mg·h/L, daily from day −6 to −2) and cyclophosphamide (14.5 mg/kg daily i.v. from days −6 to −5), as well as total body irradiation on day −1 (all received single-fraction 2 Gy, except Case 9 who received 4 Gy divided into two fractions). For GVHD prophylaxis, the patients received PTCy (cyclophosphamide 50 mg/kg daily i.v. on days +3 and +4) and then, from day +5, cyclosporine (for nine months by adjusting the serum level at 200–300 ng/mL during the first month post-transplantation and at 150–250 ng/mL thereafter) and mycophenolate mofetil (500 mg orally twice daily until day +30) were administered. Ursodiol (until day +100) was given to prevent sinusoidal obstruction syndrome. Baktar (for nine months after haploidentical PBSCT), valacyclovir (until day +30), entecavir (for one year after haploidentical PBSCT in Cases 7, 9, 11, and 12, all who were serologically positive for anti-hepatitis B core antibody), and letermovir (until day +100) were provided to prevent post-transplantation pneumocystis infection or reactivations of herpes simplex, hepatitis B, and cytomegalovirus, respectively.

## Figures and Tables

**Table 1:** Summary of the clinical characteristics of the patients.

Case No.	Age (y/o) ^a^, Sex	AA Severity ^b^	Preceding Disorder	Prior Treatment before Transfer	Adverse Events at Transfer ^c^	Salvage Treatment	Response of AA ^d^	Subtype of AA
1	19.5, male	moderate	IBD at 19 y/o	Sulfasalazine, Pred	Refractory AA	CSA for 22 months	CR > 6 years	IBD
2	2.5, female	severe	HLH at 2 y/o	rATG, CSA	Relapsed AA at 10.5 y/o	W&W	VGPR > 40 months	*SAMD9L* mutation
3	14, female	severe	No	rATG, CSA	Alopecia areata at 15.5 y/o	CSA for 8 months	CR > 9 years	Alopecia areata
4	13.5, male	severe	No	rATG, CSA	Relapsed AA at 15 y/o	CSA for 2.5 years	CR > 6 years	Idiopathic
5	10.5, male	severe	No	rATG/CSA x 2, herbs	Refractory AA	MUD-BMT	CR > 9 years	Idiopathic
6	17.5, female	severe	No	MSD-PBSCT	Evans syndrome at 19.5 y/o	Pred, CSA, MMF	CR > 8 years	Evans syndrome
7	10, male	severe	No	rATG/CSA x2	Refractory AA	Haplo-PBSCT	CR > 7 years	Idiopathic
8	9, male	severe	No	herbs	Refractory AA	Haplo-PBSCT	CR > 32 months	Idiopathic
9	7, male	severe	No	rATG/CSA/EPAG	Refractory AA	Haplo-PBSCT	CR ^e^ > 26 months	*SAMD9L* mutation
10	14, female	severe	No	CSA/EPAG	Refractory AA	Haplo-PBSCT	CR > 23 months	Idiopathic
11	4, male	severe	Hepatitis at 3.5 y/o	rATG/CSA/EPAG	Refractory AA	Haplo-PBSCT	CR >22 months	Hepatitis
12	11, female	severe	No	CSA/EPAG	Refractory AA	Haplo-PBSCT	CR > 4 months	Idiopathic

^a^. Number indicates the age (in years) at diagnosis of AA. ^b^. The patient, who was diagnosed with severe AA, had at least two peripheral cytopenias: (1) absolute neutrophil count < 0.5 × 10^9^/L; (2) platelet count < 20 × 10^9^/L; (3) hemoglobin < 8 g/dL; plus hypocellular bone marrow. Case 1 presented with gradually progressive pancytopenia during a period of about 10 months before initiating cyclosporine treatment when the peripheral blood counts revealed: (1) an absolute neutrophil count of 1.16 × 10^9^/L; (2) a platelet count of 108 × 10^9^/L; (3) hemoglobin 6.8 g/dL; plus hypocellular bone marrow. ^c^. Refractory AA indicates fulfillment of AA disease criteria at transfer after initial therapy. Relapsed AA indicates initial improvement of cytopenias after first-line therapy but subsequent return to AA disease criteria. Alopecia areata indicates appearance of bald spots of varying sizes on the scalp. Evans syndrome indicates development of immune thrombocytopenia and Coombs’ positive hemolytic anemia. ^d^. Response of AA is categorized as complete response (CR; hemoglobin ≥ 10 g/dL, absolute neutrophil count ≥ 1 × 10^9^/L, and platelets ≥ 100 × 10^9^/L) or very good partial response (VGPR; hemoglobin ≥ 8 g/dL, absolute neutrophil count ≥ 0.5 × 10^9^/L, and platelets ≥ 50 × 10^9^/L), with duration determined from when salvage treatment was initiated. ^e^. Case 9 developed Graves’ disease 10 months post-transplantation [18]. Abbreviations: AA, aplastic anemia; BMT, bone marrow transplantation; CSA, cyclosporine; EPAG, eltrombopag; Haplo, HLA-haploidentical; HLH, hemophagocytic lymphohistiocytosis; IBD, inflammatory bowel disease; MMF, mycophenolate mofetil; MSD, HLA-matched sibling donor; MUD, HLA-matched unrelated donor; PBSCT, peripheral blood stem cell transplantation; Pred, prednisolone; rATG, rabbit anti-thymocyte globulin; W&W, watch and wait; y/o, years of age.

**Table 2:** AA-risky and-protective *HLA* alleles among 10 patients (Cases 1–2, 4, 6–12).

Risky *HLA* Alleles [22,23]	Patient Carrying Risky *HLA* Alleles	Protective *HLA* Alleles [22]	Patient Carrying Protective *HLA* Alleles
HLA-A*02	Cases 6, 8, 9, 10	HLA-DRB1*03:01	Case 11
HLA-DRB1*0407	none	HLA-DRB1*04:06	none
HLA-DRB1*15	Case 1	HLA-DRB1*08:02	none
HLA-DRB1*15:01	Case 4	HLA-DRB1*13:01	Case 11
HLA-DQB1*06:02	Case 4	HLA-DRB1*13:02	none
		HLA-DRB1*14	Case 4

Associations of *HLA* alleles in Asian and Chinese Han populations [22,23].

**Table 3:** Characteristics of haploidentical PBSCT.

Case No.	Time from AA Diagnosis to HSCT	Donor (Age, y/o)	TNC Infused (10^8^/kg)	CD34^+^ Cells Infused (10^6^/kg)	CD3^+^ Cells Infused (10^8^/kg)	ABO^Rh^ D—R	CMV Status D—R	Neutrophil Engraftment ^a^	Red Cell Engraftment ^b^	Platelet Engraftment ^c^	Complete Response ^d^
7	17 months	Father (48)	12.9	10.8	3.32	O^pos^—B^pos^	pos—pos	Day +16	Day +1	Day +13	Day +20
8	3 months	Father (45)	13.5	12.6	2.92	B^pos^—O^pos^	neg—pos	Day +13	Day +131	Day +16	Day +158
9	10 months	Father (49)	10.8	14.0	1.29	O^pos^—B^pos^	pos—pos	Day +14	Day +11	Day +16	Day +18
10	60 months	Mother (46)	9.8	9.8	0.67	O^pos^—B^pos^	neg—pos	Day +18	Day +25	Day +23	Day +77
11	17 months	Brother (16)	18.0	16.6	4.07	O^pos^—O^pos^	pos—pos	Day +13	Day +7	Day +17	Day +17
12	48 months	Father (47)	19.8	8.8	5.64	O^pos^—B^pos^	pos—pos	Day +17	Day +9	Day +22	Day +77

^a^ Neutrophil engraftment was defined as an absolute neutrophil count of ≥0.5 × 10^9^/L for three consecutive measurements on different days after post-transplantation nadir. ^b^ Red cell engraftment was counted as days from last packed red cells transfusion. ^c^ Platelet engraftment was defined as a platelet count ≥ 20,000 × 10^9^/L for three consecutive measurements on different days in the absence of platelet transfusion for seven consecutive days. ^d^ Complete response was defined as the reach and persistence of hemoglobin ≥ 10 g/dL, absolute neutrophil count ≥ 1 × 10^9^/L, and platelets ≥ 100 × 10^9^/L in the absence of transfusion. Abbreviations: AA, aplastic anemia; CMV, cytomegalovirus; D—R, donor—recipient; HSCT, hematopoietic stem cell transplantation; neg, negative; pos, positive; TNC, total nucleated cells; y/o, years of age.
